# The Expanding Burden of Elevated Blood Pressure in China: Evidence From Jiangxi Province, 2007–2010

**DOI:** 10.1097/MD.0000000000001623

**Published:** 2015-10-02

**Authors:** Gang Xu, Junxiu Liu, Shiwei Liu, Haiming Zhou, Olubunmi Orekoya, Jie Liu, Yichong Li, Ji Tang, Chunlian Zhou, Jiuling Huang

**Affiliations:** From the Department of Preventive Medicine, School of Basic Medicine, Jiangxi University of Traditional Chinese Medicine, Nanchang, China (GX, JH); Department of Epidemiology and Biostatistics, Arnold School of Public Health, University of South Carolina, Columbia, South Carolina, USA (JL, OO); National Center for Chronic and Non-communicable Disease Control and Prevention, Chinese Center for Disease Control and Prevention, Beijing, China (SL, YL); Department of Statistics, University of South Carolina, Columbia, South Carolina, USA (HZ); Division of Chronic Disease Control and Prevention, Jiangxi Province Center for Disease Control and Prevention, Nanchang, Jiangxi, China (JL); Chinese Preventive Medicine Association, Beijing, China (JT); and Department of Nosocomial Infectious Prevention and Control, Beijing Friendship Hospital, Capital Medical University, Beijing, China (CZ).

## Abstract

Elevated blood pressure (BP) as a risk factor accounts for the biggest burden of disease worldwide and in China. This study aimed to estimate attributed mortality and life expectancy (LE) to elevated BP in Jiangxi province between 2007 and 2010.

BP and mortality data (2007 and 2010 inclusive) were obtained from the National Chronic Diseases and Risk Factors Surveillance Survey and Disease Surveillance Points system, respectively. Population-attributable fraction used in comparative risk assessment of the Global Burden of Disease study 2010 were followed to quantify the attributed mortality to elevated BP, subsequently life table methods were applied to estimate its effects on LE. Uncertainty analysis was conducted to get 95% uncertainty intervals (95% uncertainty interval [UI]) for each outcome.

There are 35,482 (95% UI: 31,389–39,928) and 47,842 (42,323–53,837) deaths in Jiangxi province were caused by elevated BP in 2007 and 2010, respectively. 2.24 (1.87–2.65) years of LE would be gained if all the attributed deaths were eliminated in 2007, and increased to 3.04 (2.52–3.48) in 2010. If the mean value of elevated BP in 2010 was decreased by 5 and 10 mm Hg, 5324 (4710–5991) and 11,422 (10,104–12,853) deaths would be avoided, with 0.41 (0.37–0.48) and 0.85 (0.71–1.09) years of LE gained, respectively.

The deaths attributable to elevated BP in Jiangxi province has increased by 35% from 2007 to 2010, with 0.8 years of LE loss, suggesting the necessity to take actions to control BP in Chinese population.

## Abstract 2

Supplemental Digital Content is available in the text

## INTRODUCTION

Elevated blood pressure (BP), as a major risk factor, has been associated with raised risk of many chronic diseases such as cardiovascular disease (CVD)^1–3^ and chronic kidney disease (CKD).^4^ Elevated BP is also linked to lower life expectancy (LE).^5^ According to the comparative risk assessment (CRA) of the Global Burden of Disease (GBD) study, elevated BP accounted for 9.4 million deaths and 7.0% of disability-adjusted life years in 2010 worldwide, and the attributed burden of disease increased in rank from the fourth in 1990 to the first in 2010 among 20 leading single risk factors.^6^ While in China, approximately 2.0 million deaths and 9.3% of disability-adjusted life years were attributable to elevated BP, and the corresponding burden from elevated BP ranked the first among the 67 single risk factors examined in 2010, followed by tobacco smoking.^7^ In general, 85% of annual deaths in China are attributable to noncommunicable diseases (NCDs) and among them CVDs ranks number 1, accounting for 45% of total mortality.^7,8^

Previous national surveys conducted in China showed the prevalence and absolute numbers of hypertension have increased dramatically during the past decades.^3,9–11^ The prevalence of hypertension among Chinese adults has sextupled from 5% in 1959 to more than 30% in 2010,^9,11^ and the estimated number of hypertension cases has 10-fold from 30 million in 1960 to 300 million in 2010.^3,11^ However, elevated BP is the most preventable risk factor for CVD.^7,12^ Little research has examined the role of elevated BP as a risk factor for disease burden in China, especially at the provincial level. In addition, no study has examined changes in LE attributable to elevated BP in China.

This study aimed to estimate the attributed mortality to elevated BP and subsequently the LE loss in Jiangxi province in 2007 and 2010, using risk factor data from the Chinese National Chronic Diseases and Risk Factors Surveillance Survey (CDRFSS) and the same years’ mortality data from the Chinese Disease Surveillance Points (DSP) system,^13^ and to estimate the avertable deaths or obtainable LE by reducing the epidemic level of this risk factor at different counterfactual scenarios. All the estimates were made under the hypothesis that the present BP distribution in study years or the counterfactual BP distribution has remained stable for sufficient exposure times.

## METHODS

### Study Population

Jiangxi province, located in southeast China, is situated at 24°29′14″–30°04′41″ north latitude and 113°34′36″–118°28′58″ east longitude, and spans from the banks of the Yangtze River in the north into hillier areas in the south and east. The altitude of the residential area in Jiangxi province is 50–500 m above mean sea level, the mean annual air temperature is 16.3–19.5°C, and the mean annual precipitation is 1341–1943 mm. The census 2010 data showed that the population of Jiangxi province was approximately 44.57 million, with 72.06% of the population engaged in agriculture (nationwide: 70.13%). The urbanization rate was 43.75% (nationwide: 50.27%). The proportion of the population ≥65 years of age was 7.60% (nationwide: 8.92%), in 2010. In contrast to the country, there has a larger proportion of agriculture population in Jiangxi province with low urbanization, and less aging processes.

## DATA SOURCES

### Exposure Data of Elevated Blood Pressure

The data of BP values among adults 18 years of age or older were retrieved from Chinese CDRFSS 2007 and 2010, which were independently carried out in the surveillance points (1 point corresponds to 1 county or district) of the DSP system.^10,11,14–16^ All participants were randomly selected by a multistage stratified random sampling method in each point (in total of 5 points in Jiangxi province); the sampling procedure and sample size were described elsewhere.^10,11^ According to a standard protocol, data collection was conducted by trained staff in examination centers at local health stations or community clinics which are convenient and accessible for the participants. Finally, 1551 and 2771 participants living locally more than 6 months in 2007 and 2010, respectively, were included in our analysis. BP was measured by trained and certified research staffs 3 times using standardized electronic sphygmomanometer after a 5–15 minutes sitting rest both in 2007 and 2010. The average value of the last 2 measurements was calculated as the final value. Additionally, in order to make the samples more representative of the total province, the sample weighting and sex- and age-specific structure adjustment were applied on the sample population on the basis of the whole population across the province.^17^ Because the CDRFSS was conducted among persons ≥18 years with few more than 69 years, data from participants in the age groups 70–74, 75–79, and ≥80 were combined with age group 65–69, and the same values were utilized for them.

All enrolled participants in the CDRFSS gave written and informed consent for participation. The National Health and Family Planning Commission (NHFPC, previously Ministry of Health) of China and the Ethics Committee of the Chinese Center for Disease Control and Prevention approved the implementation of CDRFSS and mortality surveillance through the DSP system. The records/information of participants was deidentified prior to analysis.

Being consistent with the methodology of CRA framework in the GBD study,^6,18^ BP was defined as systolic blood pressure (SBP) in mm Hg, which was treated as a continuous variable in our study.

### Mortality Data

Mortality data for 2007 and 2010 by underlying causes and sex-age were extracted from the DSP system^13^ which includes 5 surveillance points in Jiangxi province.^19,20^ Due to the built-in weaknesses of surveillance system, such as completeness of reporting and accuracy of cause of death, we applied the well-established methods for the adjustments on under-reporting, garbage code redistribution of causes of death and provincial representativeness.^21^

### Population Data

The sex-age population of each surveillance point in 2007 and 2010 was extracted from National Bureau of Statistics of China.

### Health Outcomes and the Relative Risk (*RR*)

In accordance with CRA in the GBD study,^6^ ischemic heart disease (IHD), ischemic stroke (Istroke), hemorrhagic stroke (Hstroke), rheumatic heart disease (RHD), endocarditis, cardiomyopathy, and myocarditis (ECM), aortic aneurysm (AA), hypertensive heart disease (HHD), atrial fibrillation, peripheral vascular disease, and other circulatory diseases (APO), and CKD were identified as health outcomes etiologically associated with elevated BP as a risk factor. GBD team calculated the *RR* per 10 mm Hg increasing in SBP by age and by each health outcome using meta-analysis based on results from large cohort studies, randomized controlled trials, consistently adjusted for confounding factors, and accounted for regression dilution bias based on serial BP measures over time.^6,18^

## STATISTICAL ANALYSIS

### Conceptual Framework of CRA^22^

The CRA is a systematic evaluation of the changes in population health which would result from modifying the population distribution of exposure to a risk factor or a group of risk factors. In particular, the burden of disease due to the observed exposure distribution in a population is compared with the burden from a hypothetical distribution or series of distribution. Categorical attribution and counterfactual analysis as 2 traditional causal attribution are widespread used, in which categorical attribution means 100% of the event is attributed to the single cause or group of causes (in fact it is seldom for many diseases usually have multiple causes), while counterfactual analysis is estimated by comparing the current or future levels of the summary measure with the levels that would be expected under some alternative hypothetical scenario including the absence of or reduction in risk factor, which has widespread used in epidemiological study. The basic statistic obtained in counterfactual analysis is the population-attributable fraction (PAF) defined as the proportional reduction in disease or death that would occur if exposure to the risk factor were reduced to zero or counterfactual distribution, *ceteris paribus*. Commonly counterfactual distributions are determined based on theoretical, plausible, feasible, or cost-effective minimum risk according to study needs.

### Mortality Attributable to Elevated Blood Pressure

In our analysis, PAF was calculated to estimate the burden attributable to excess SBP by comparing the present distribution of SBP to the theoretical minimum SBP and other counterfactual SBP distribution for each specified sex-age group, year, and 9 SBP-related health outcomes, respectively.^6,23^ The formula used was: 
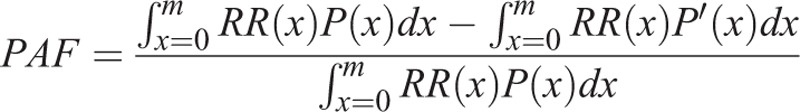


where p(x) is the population distribution of SBP, p’(x) is the theoretical minimum SBP distribution or counterfactual distribution of SBP, *RR*(x) is the relative risk at SBP level x, and m is the maximum SBP level. The attributed mortality for each disease causally associated with elevated SBP was calculated by multiplying the PAF with the observed deaths according to specific sex and age group, and then the total attributable disease mortality was summed as the whole mortality attributable to SBP.

### Effects of High Blood Pressure on LE

We calculated the LE for each year using standard life table methods, based on the observed age-specific mortality (LEyt), as well as the alternative age-specific mortality by removing the deaths attributable to excess SBP (LEyr). The difference between the 2 sets of LEs at birth measures the LE gains (LEyr–LEyt). Similarly, LE shifts for the current distributions of BP changed to an alternative scenario can be calculated using the above proposed methods.

### Alternative Blood Pressure Distributions

We applied 3 different counterfactual SBP distributions to track the effects of elevated BP on mortality and LE in Jiangxi province by comparing each of them to that in 2010, treated as current distribution: the mean of SBP for each sex-age group was decreased to the theoretical minimum distribution of 115 mm Hg with standard deviation (SD) of 6 mm Hg, the mean of SBP for each sex-age group was decreased by 5 mm Hg with unchanged SD, and the mean of SBP for each sex-age group was decreased by10 mm Hg with unchanged SD.

### Uncertainty Analysis

Considering several forms of uncertainty resulting from incomplete information, potential biases or heterogeneity of input data, or the analysis models used in the estimate of disease burden,^24^ Monte Carlo simulation techniques were applied in a similar way as before to present uncertainty ranges around point estimates reflecting the main sources of uncertainty.^21^ The @RISK software version 6 for Excel^25^ allows multiple recalculations of a spreadsheet. We defined the *RR* and SBP value as the input variables, and the PAF, attributed deaths and LE as the output variables. For the input variables we specified a log-normal distribution for the *RR* and normal distribution for the SBP value. For each of the output variables, 95% uncertainty intervals (95% uncertainty interval [UI]) were calculated bounded by the 2.5th and 97.5th percentiles of the 2000 iteration values generated.

## RESULTS

Table 1 shows the distribution of SBP (mean and SD) according to 2007 and 2010 CDRFSS by sex and age. In general, SBP is increasing with age, and the mean SBP in 2010 is higher than that in 2007. In 2007, young male (<55) has higher mean SBP than young female (<55), while male has higher mean SBP than female in 2010 across all age strata except age group of 55–59.

**TABLE 1:**
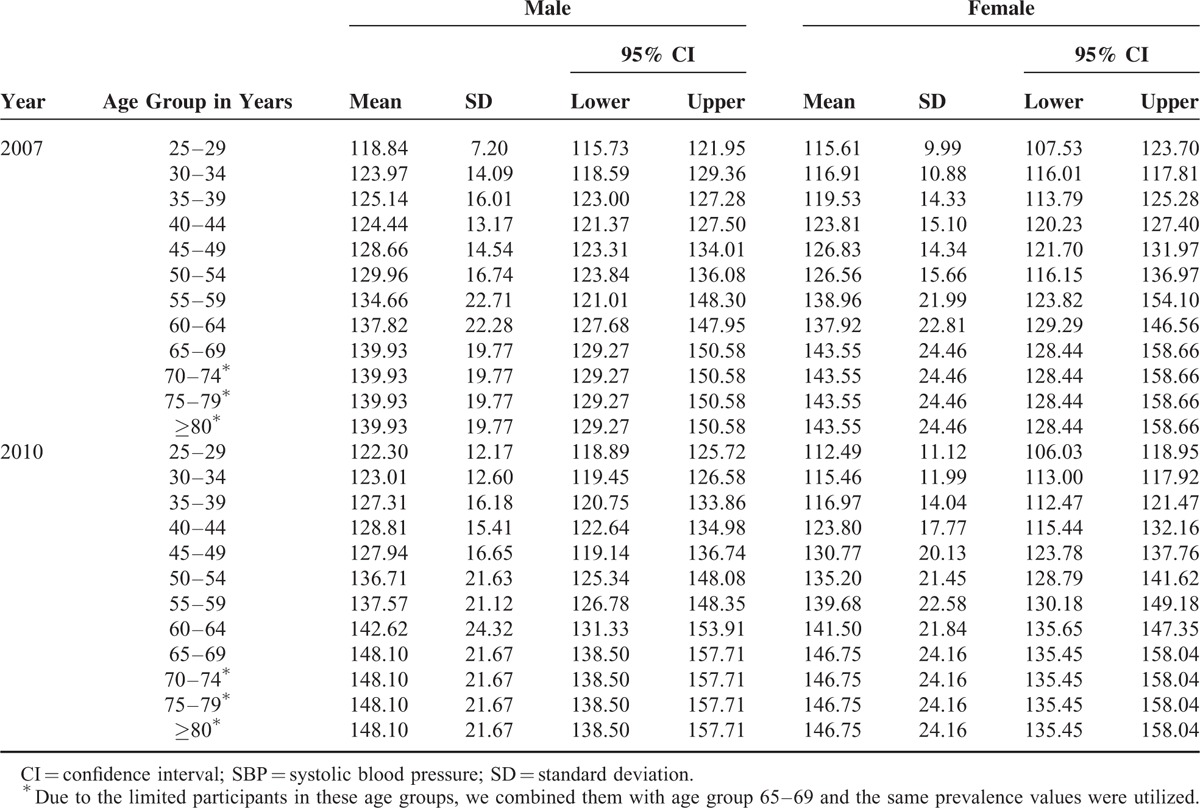
Mean SBP and SD in Jiangxi Province of China by Age-Sex, 2007 and 2010

The *RR*s and 95% confidence intervals (CIs) for selected health outcomes by age are presented in Appendix Table 1, http://links.lww.com/MD/A430. HHD had the highest risk attributable to SBP, followed by stroke and IHD in comparison to other health outcomes.

Table 2 shows the overall and sex specific PAFs for 9 selected health outcomes between 2007 and 2010. Generally, the PAFs of IHD, Istroke, Hstroke, RHD, ECM, HHD, APO, CKD attributable to elevated SBP have all been significantly increased, with the same trend for males (for females, the fraction for AA was increased). In total, the PAF of the 9 outcomes increased from 51.6% in 2007 to 55.9% in 2010. Attributable fractions for all related health outcomes were higher in female than male in 2007; however, inverse findings were observed in 2010. Overall, 35,482 (95% UI, 31,389–39,928) deaths, accounting for 16.6% (95% UI, 13.7–19.1%) of total deaths in Jiangxi province in 2007, and 47,842 (95% UI, 42,323–53,837) deaths, accounting for 22.0% (95% UI, 17.8–25.4%) of total deaths in 2010, were attributable to elevated SBP. The majority of the attributed deaths resulted from stroke (Istroke and Hstroke, especially Hstroke), followed by IHD. The LE gains for elimination of attributed deaths to elevated SBP were significantly increased and were displayed in Table 2 for 2007 and 2010, respectively.

**TABLE 2:**
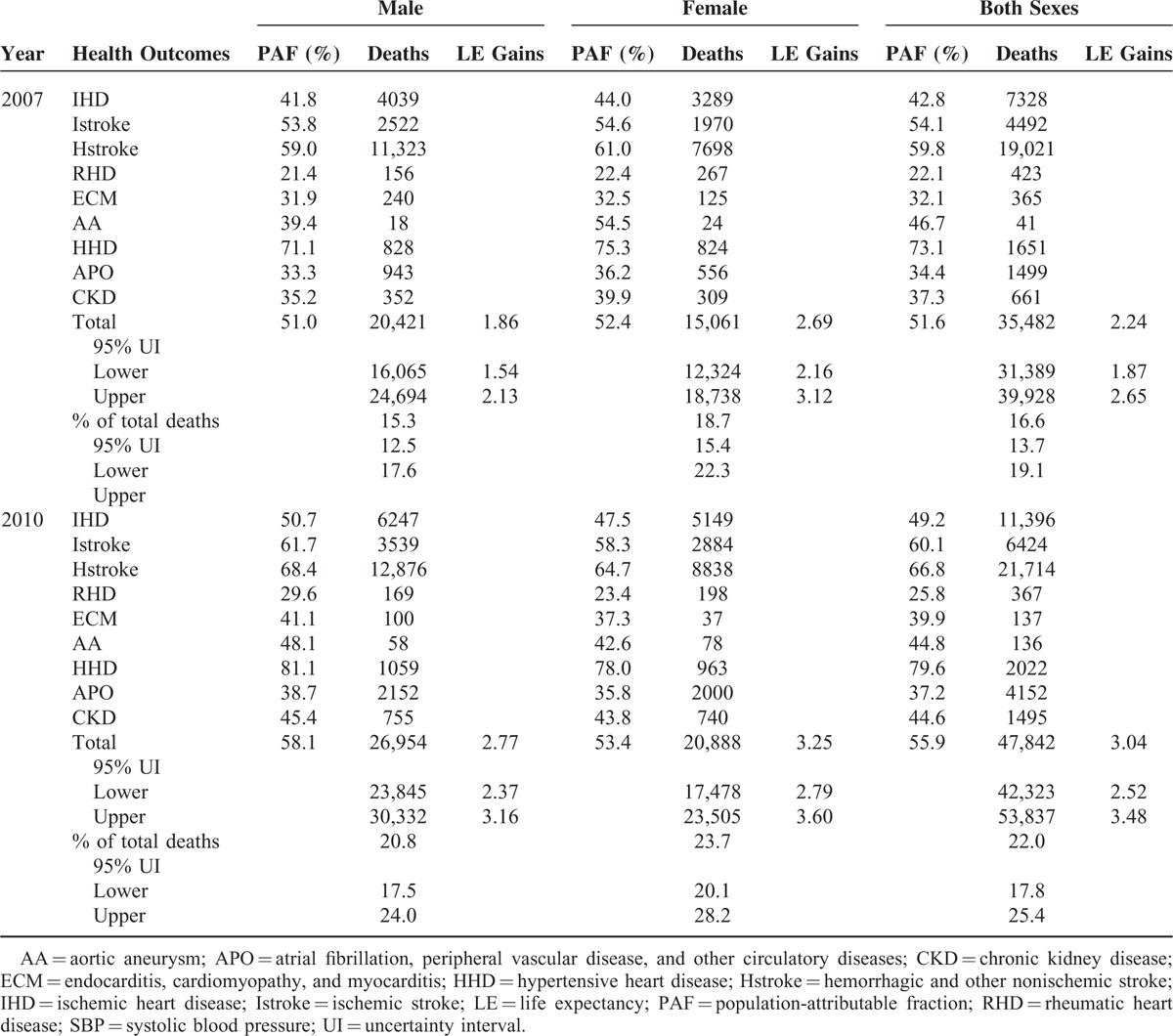
PAF (%), Deaths Attributable to Elevated SBP and LE Gains Attributable to SBP Reduction by Age and Sex, Jiangxi Province of China, 2007 and 2010

**FIGURE 1:**
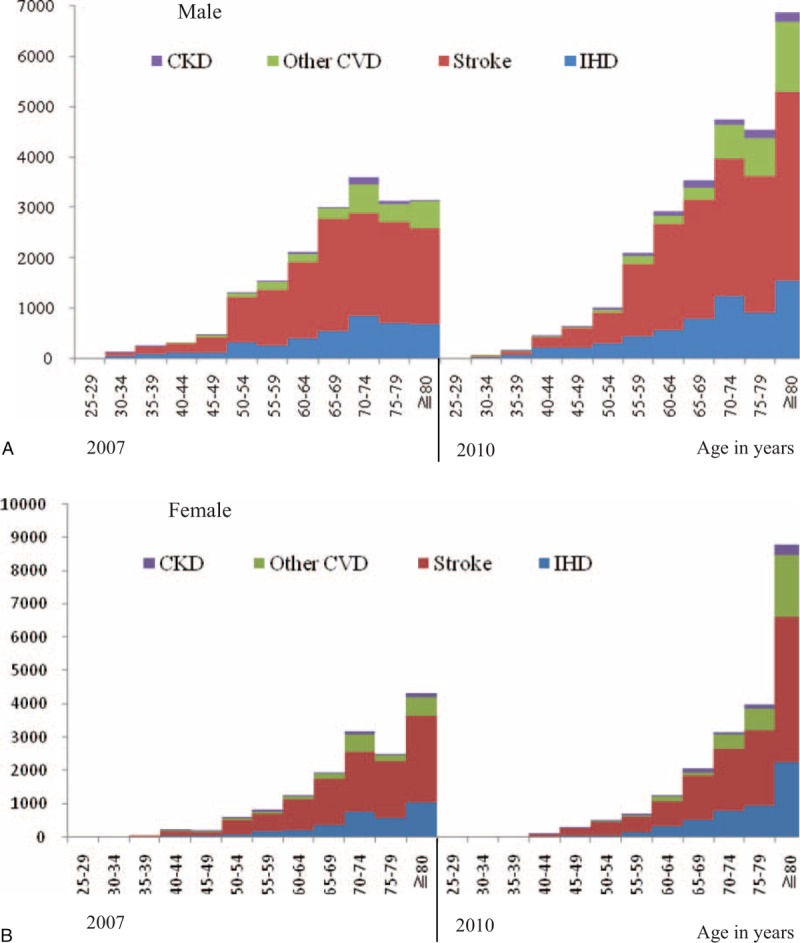
Deaths attributable to elevated SBP by age and sex, Jiangxi province of China in 2007 and 2010.

Figure 1 shows the deaths attributable to elevated SBP across sex and age groups between 2007 and 2010. In total, the deaths attributable to elevated SBP were higher in 2010 than 2007 and higher in older age groups for both males and females. Stroke and IHD took the majority of deaths attributable to elevated SBP in both 2007 and 2010. A larger proportion of deaths in 2007 occurred in the 70–74 age group in both males and females, while in 2010 both males and females have largest proportion of death at >80 years old.

**FIGURE 2:**
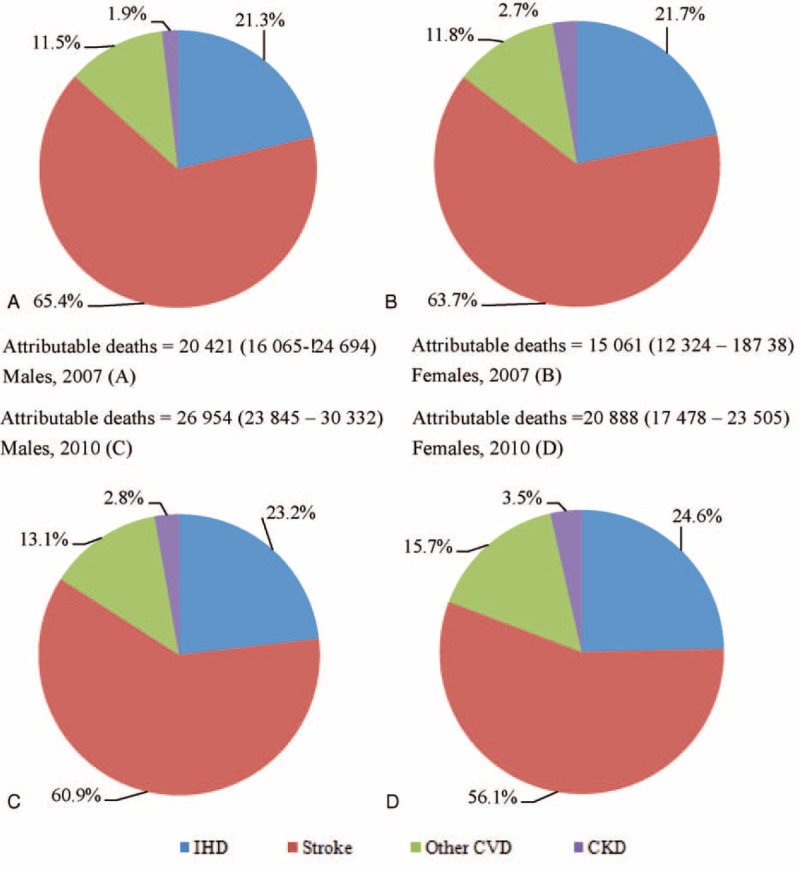
Deaths attributable to elevated SBP by sex and conditions, Jiangxi province of China, 2007 and 2010.

Figure 2 shows that the proportion of deaths caused by stroke, IHD, other CVD and CKD. Stroke (Istroke and Hstroke, more than 55%) took the largest proportion both in 2007 and 2010, followed by IHD (more than 20%). IHD accounted for 21.3% and 21.7% of deaths in 2007 and increased to 23.2% and 24.6% in 2010, respectively, for males and females. However, the percentage of death from stroke declined in 2010 compared to 2007. CKD accounted for 1.9% and 2.7% of deaths in 2007 and increased to 2.8% and 3.5% in 2010 for males and females, respectively.

Table 3 shows the avoided number of deaths, years of LE gained and their corresponding 95% UI by assuming that the mean of SBP for all sex-age groups was decreased by 5 or 10 mm Hg in 2010, respectively.

**TABLE 3:**
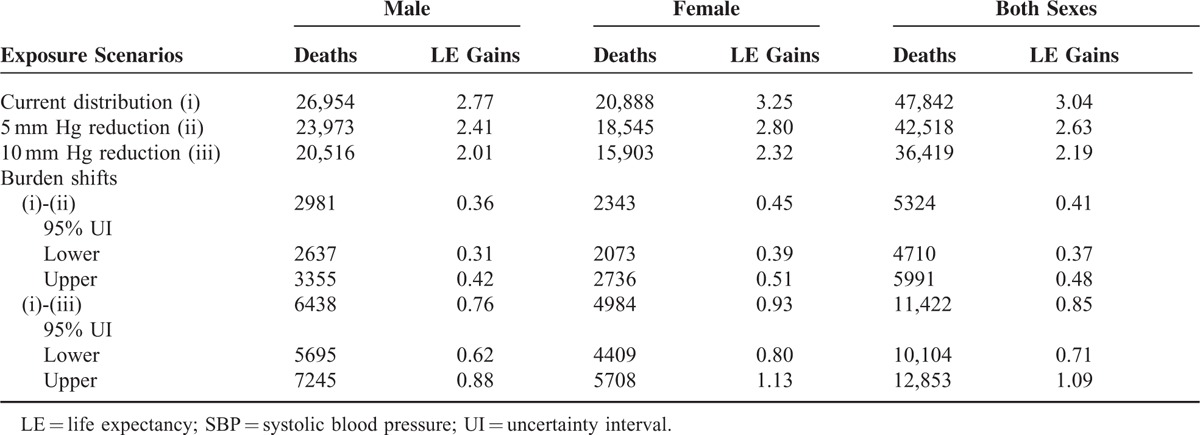
LE Shifts at Different Counterfactual Scenarios of SBP by Sex Comparing With Current Distribution in 2010, Jiangxi Province of China

## DISCUSSION

This study demonstrated the expanding elevated SBP-related disease burden from 2007 to 2010 in Jiangxi province of China, which mainly embodies the increasing value of the fractions and the number of deaths attributable to elevated SBP. Although overall mortality rate in Jiangxi province declined between 2007 and 2010, the number of deaths attributable to elevated SBP in 2010 was higher than in 2007. The attributable fraction of overall elevated SBP-related health outcomes for females was slightly higher than that for males in 2007 while inverse result was found in 2010. However, the number of attributable deaths for males was greater than for females in both 2007 and 2010. These findings of increased mean of SBP and decreased all-cause mortality rate in Jiangxi province are consistent with national surveillance data from the CDRFSS^10,11^ and DSP system^19,20^ across the country in 2010 comparing with 2007. Hence, our finding based on the data from Jiangxi province mirrored that the disease burden attributable to elevated SBP were expanding in the entire country of China. As a result of rapid economic growth, improved health system, and increased health awareness, the age-standardized mortality rate in China has declined by 32% from 1990 to 2010, with much for the infectious diseases and maternal and prenatal conditions (62%). Conversely, the proportion of chronic disease has increased by 14% and the burden of disease attributable to physiological risks and behavioral risks has substantially increased.^7^

Previous studies have showed that elevated BP is positively associated with higher social economy,^26^ coastal areas,^27^ north areas with lower temperature,^28^ and higher altitude areas.^29^ The disease burden attributable to elevated SBP in Jiangxi province, as an inland, southeast, low altitude area with middle social economy development comparing with most of other provinces (ranked 25th for gross domestic product per capita in 2013), reflects higher burden for most of other provinces in China.

The 2000 GBD study^18^ showed that 49% of IHD, 62% of stroke, and 76% of hypertensive disease were attributable to elevated SBP (>115 mm Hg) worldwide in the year 2000. Approximately 7.1 million deaths accounting for 12.8% of the total were due to nonoptimal BP. The 2010 GBD study^6^ showed that 9.4 million global deaths (∼17.8% of all deaths) were attributable to elevated BP. Comparatively the burden of disease attributable to elevated BP for China is much greater than for all over the world. From 1990 to 2010, the attributable number of deaths from elevated BP was significantly increased from 1.3 (16.6%) to 2.0 million (24.6%).^7^ In our study, elevated SBP caused 35,482 and 47,842 deaths in 2007 and 2010, respectively, which accounted for 16.6% and 22.0% of total deaths in Jiangxi province. Hence, comparing to the entire country of China, the burden of disease attributable to elevated SBP increased faster in Jiangxi province. Similar to the GBD study, our study showed that 49.2% of IHD, 60.1% of ischemic stroke, 66.8% of hemorrhagic stroke and 79.6% of HHD were attributable to elevated SBP (>115 mm Hg). Therefore, our estimates of the mortality burden due to elevated SBP in Jiangxi province are plausible and comparable to previous studies. Our findings further support the needed effort to reduce elevated BP in China.

CVDs account for about 45% of annual deaths which is the leading cause of death in China.^7,8^ This study showed that CVDs accounted for more than 95% of deaths attributable to elevated BP for both males and females in both 2007 and 2010, the majority of which is stroke (more than 55%), followed by IHD and other CVDs. This study also found that the proportion of stroke deaths attributable to elevated SBP decreased from 2007 to 2010, while IHD and other CVDs increased. This increasing trend for IHD mortality and slight declining for stoke mortality was also observed in the results of age-standardized mortality rate from 1991 to 2013 in China based on the DSP system (data not presented here). Controlling BP is a most direct and effective measure to decrease CVDs.

The twelfth 5-year plan for national economic and social development of China, initiated in 2011,^30^ firstly proposed to increase LE by 1 year, from 2010s 73.5 years to 2015s 74.5 years. Since then, each local government has developed programs to the greatest extent to increase LE in its populations by 1 year. In the present study, we estimated for the first time the effects of reducing BP on LE in China using Jiangxi province data, which showed that reduction of SBP in 2010 to the theoretical minimum distribution would have increased the LE by 3.04 years (2.77 for males and 3.25 for females). We also tracked the effects of elevated BP on LE shifts in Jiangxi province at different counterfactual SBP distributions, compared with 2010s distribution. It was assumed that the means of SBP in each sex-age group decreased by 5 and 10 mm Hg would increase LE by 0.41 and 0.85 years, respectively. These estimates on burden of disease attributable to risk factors are expected to be applied to evaluate public health performance and to guide health intervention and policy-making.

Some limitations have to be laid out. First, the study population might not be representative of the entire province since in each CDRFSS the participants were enrolled by multistage stratified random cluster sampling across the country. However we employed both the sample weighting, and sex- and age-specific structure adjustment on the sample population to be best representative of the whole population across the province. Second, the *RR* values used in our study were directly obtained from CRA of the GBD study on the basis of pooled global data. Though, it is reasonable to assume that they are valid and robust since they have been widely used in China^7^ and other developing countries.^31^ Third, the calculation of LE gains was based on 2 cross-sectional CDRFSS and the BP values might vary for seasonal fluctuation^32^ and other different environmental factors.^33^ Hence the comparability between 2010 and 2007 might be compromised to some extent. Moreover, the mean estimates of SBP might not be stable due to the relatively small sample sizes at provincial level in the 2 CDRFSS.^10,11^ The same concern exists for the mortality from DSP system since only 5 surveillance points involved.^19,20^ But, fortunately each field site of the 2 CDRFSS is consistent with the DSP, which might greatly reduce the estimation error of excess deaths in this study.

In conclusion, China has gone through a rapid demographic change in the past decades, and unhealthy lifestyles associated with socioeconomic improvement have crept up resulting in a greater burden of noncommunicable diseases. The potential lifestyle factors independently and jointly contributing the increase of BP include high salt intake, obesity, physical inactivity, tobacco smoking, diet low in vegetables and fruits, excessive alcohol use.^6,7^ The diversity and strength of the evidence is much greater than other lifestyle factor.^34^ International experiences have proved good effectiveness on the prevention of CVD by implementing lifestyle interventions, for example the PREDIMED trial in Spain,^35^ the Montana program in USA.^36^ There is an urgent need for the Chinese government to take action to curb the epidemic of unhealthy lifestyles in the population, meanwhile strengthen the management of hypertensive patients. Preventing or reducing elevated BP behaviors should be a priority within China's healthcare reform agenda in order to reduce the BP-related disease burden.
